# In Situ Synthesis of Bacterial Cellulose-Supported CoAl-Layered Double Hydroxide as a Peroxymonosulfate Activator for Enhancing the Removal of Tetracycline

**DOI:** 10.3390/biom15091283

**Published:** 2025-09-05

**Authors:** Xiuzhi Bai, Yongsheng Du, Zhongxiang Liu, Jing Cheng, Jie Yang, Ying Li

**Affiliations:** 1 School of Chemistry and Chemical Engineering, Henan Institute of Science and Technology, Xinxiang 453003, China; bai_xiuzhi@hist.edu.cn (X.B.); duys123@stu.hist.edu.cn (Y.D.); liuzhongxiang9527@stu.hist.edu.cn (Z.L.); yangj61@stu.hist.edu.cn (J.Y.); 2 Hunan Key Laboratory of Food Safety Science Technology, Technology Center of Changsha Customs, Changsha 410004, China

**Keywords:** bacterial cellulose, layered double hydroxide, peroxymonosulfate, antibiotics, catalysis

## Abstract

This study employed the hydrothermal coprecipitation method to grow CoAl-layered double hydroxide (LDH) onto bacterial cellulose (BC) in situ, successfully preparing the CoAl-LDH@BC composite. This composite was then used to activate peroxymonosulfate (PMS) for tetracycline (TC) degradation. According to the results, the CoAl-LDH@BC/PMS system demonstrated a remarkable removal efficiency of 99.9% for TC within 15 min. Moreover, the influencing factors of catalyst dosage, PMS dosage, TC concentration, reaction temperature, initial pH, and inorganic ions were evaluated. Notably, the system demonstrated broad-spectrum contaminant removal capabilities, which could simultaneously eliminate more than 99.7% of oxytetracycline hydrochloride (TCH) and 87.9% of ciprofloxacin (CFX) within 20 min. Additionally, the removal rates for several dyes reached more than 95.7% in 20 min. Phytotoxicity assessment (using mung bean seeds) confirmed a significant reduction in the biotoxicity of post-treatment TC solutions. The identification of TC degradation intermediates was enabled, alongside the subsequent proposal of plausible degradation pathways. Furthermore, mechanistic investigations based on radical quenching experiments revealed the coexistence of dual radical (•OH and${\;\mathrm{SO}}_{4}^{-}•$) and non-radical (^1^O_2_) oxidation pathways in the reaction of the CoAl-LDH@BC/PMS system. Overall, this research broadens the potential applications of bacterial cellulose-based porous materials and provides an innovative insight into antibiotic wastewater treatment.

## 1. Introduction

As a basic resource, water is essential for human survival and development. In recent years, water environment pollution has become increasingly serious, which has become a predominant restriction for the high-quality development of society and the construction of ecological civilization [1,2]. Specifically, environmental pollution involves the release of numerous antibiotics into the natural environment [3]. For instance, as the world’s second most widely used antibiotic, tetracycline (TC) owes its prevalence to its cost-effectiveness and potent broad-spectrum antibacterial activity. TC may destroy the ecosystem in the water environment due to its high biological toxicity and difficult degradation, which could result in water deterioration and other problems including microbial resistance, accelerating the diffusion and spread in the ecological environment [4]. Therefore, the development of an efficient and convenient technology is necessary for the removal of TC from the water environment.

Currently, TC removal methods are mainly composed of adsorption, membrane separation, coagulation, microbiological techniques, and advanced oxidation processes (AOPs) [5,6,7,8,9]. Among these methods, peroxymonosulfate (PMS)-based AOPs have garnered significant attention due to their advantages of having a low cost, high redox potential, and stability in a wide pH range [10]. Based on its asymmetric structure and low binding energy, PMS is prone to being activated by certain factors (including heat, ultraviolet light, ultrasound, or transition metals) to form the reactive oxygen species (ROS) (such as ${\;\mathrm{SO}}_{4}^{-}\text{•}$, •OH, ${\text{•}O}_{2}^{-}$, and ^1^O_2_) [11]. Functionally, heterogeneous catalysts based on Co, Fe, Cu, Ni, and Mn metals are widely used to activate PMS by transferring electrons to PMS to form ROS, and they exhibit ideal catalytic ability for the degradation of organic pollutants [12,13]. Notably, cobalt-based species have demonstrated superior performance in the PMS-based AOP field, making them highly promising activators for PMS [14]. However, the presence of residual cobalt ions (Co^2+^) may pose environmental and public health risks, which significantly hinder their further development [15]. Therefore, it is important to improve the stability of cobalt-based catalysts. Recently, layered double hydroxide (LDH) has become a focus of interest, which could be attributed to its superior stability and lower metal ion leaching versus the traditional metal catalysts during wastewater treatment [16]. Furthermore, LDH has demonstrated excellent performance in PMS activation for catalytic degradation of pollutants, which could be attributed to their ion exchange properties and diverse metal-oxygen functional groups, alongside a self-restoring layered structure for reusability [17]. However, the separation of a pure LDH catalyst is relatively difficult, and the recycling is inconvenient [18]. Moreover, the aggregation of LDHs inhibits the activation of PMS, which may reduce the degradation efficiency of pollutants [19].

In order to address these limitations, numerous carbon-based substances have been utilized as support materials to evenly disperse LDHs and minimize clumping to enhance their catalytic capacity and durability [20]. Bacterial cellulose (BC) is a three-dimensional network structure mesh of interwoven nanofibers, which is characterized by its affordable, chemically stable, and biodegradable properties [21]. Specifically, BC exhibits the characteristics of a low density and high porosity, alongside the advantages of a wide source of raw materials, being clean and renewable, and having good biocompatibility and degradability, and it has attracted extensive attention from scholars in China and abroad [22]. Notably, the remarkable features of BC could provide fundamental support and dispersion of catalytic actives as a matrix for catalyst production [23]. For example, BC was applied as a scalable carrier for the growth of Mn-MOF, and MnO/CBC was successfully employed as a catalyst to activate PMS for the degradation of TC [24]. Despite BC being an exceptional catalyst support, its application in AOPs remains underexplored.

In this work, a one-step method was presented to fabricate the CoAl-LDH@BC composite with the objective to address the separation and aggregation issues faced by conventional powdery catalysts. The CoAl-LDH film was in situ grown on BC via a one-step hydrothermal treatment and was used to activate PMS to degrade TC. Subsequently, various characterization methods were employed to characterize the prepared catalysts, followed by the investigation of the effects of certain factors, including the amount of catalyst, the amount of oxidant, the initial concentration of TC, the initial pH of the solution, the reaction temperature, and the common inorganic ions. Additionally, the catalytic mechanisms of the reaction system were deeply explored, alongside the possible degradation path of TC. Overall, this study seeks to provide a reference for the synthesis of new catalysts, alongside valuable insights into the development of organics in wastewater treatment.

## 2. Materials and Methods

### 2.1. Materials

Tetracycline (TC, C_22_H_24_N_2_O_8_, analytical standards), cobalt nitrate hexahydrate (Co(NO_3_)_2_⋅6H_2_O, ≥99.9% metals basis), aluminum nitrate nonahydrate (Al(NO_3_)_3_⋅9H_2_O, ≥99.99% metals basis), urea (H_2_NCONH_2_, ≥99.999% metals basis), anhydrous ethanol (≥99.5%, H_2_O ≤ 0.005%), isopropanol (IPA, ≥99%), L-histidine (≥99%), p-benzoquinone (p-Bq, ≥99%), potassium peroxymonosulfate (PMS, ≥42%), sodium hydroxide (NaOH, ≥97%, flakes), and hydrochloric acid (HCl, 37%) were supplied by Aladdin Chemistry Co., Ltd. (Shanghai, China). Bacterial cellulose (32 × 26 × 0.2 cm) was purchased from Tianlu Nano (Nanjing, China).

### 2.2. Preparation of CoAl-LDH@BC

**Figure 1:**
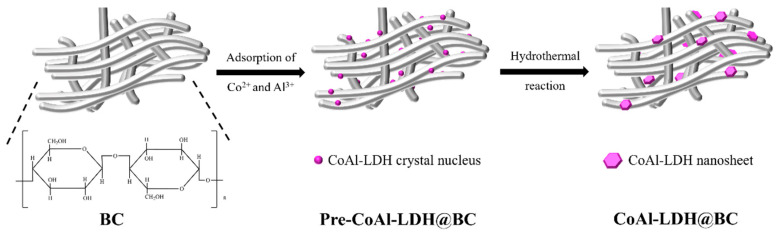
Schematic diagram of the synthetic process of the CoAl-LDH@BC composite.

Figure 1 shows a schematic diagram of the synthetic process of the CoAl-LDH@BC composite. During the preparation of CoAl-LDH@BC, the BC was initially cut into suitable-sized chunks, followed by soaking it in deionized water for 24 h. Subsequently, the BC block was soaked in 0.1 M NaOH solution and heated at 80 °C for 3 h. After repeated rinsing of soaked BC with deionized water, some residual magazines (such as bacteria and by-products) were removed to obtain the purified BC. Subsequently, Co(NO_3_)_2_⋅6H_2_O, Al(NO_3_)_3_⋅9H_2_O and urea (fixed molar ratio of 2:1:15) were dissolved in deionized water to form a pre-LDH solution, and the purified BC block was soaked in this solution overnight. Thereafter, the mixed solution was transferred to the reaction still and heated at 80 °C for 24 h. After cooling to room temperature, the mixed solution was rinsed with deionized water and anhydrous ethanol repeatedly to remove the unreacted substance. The obtained products were freeze-dried, and the CoAl-LDH@BC was finally prepared. The CoAl-LDH was prepared using an identical procedure but without the addition of BC. The total molar concentrations of Co^2+^ and Al^3+^ were adjusted to 0.01 M, 0.02 M, 0.04 M, 0.06 M, 0.08 M, and 0.10 M to obtain CoAl-LDH@BC loaded with different LDH concentrations, and the obtained samples were labeled with x-CoAl-LDH@BC (x = 0.01, 0.02, 0.04, 0.06, 0.08, and 0.10).

### 2.3. Catalyst Characterization

The Regulus 8100 field emission scanning electron microscope (FESEM) (Hitachi, Tokyo, Japan) was employed to observe the microscopic morphological characteristics, while the Bruker D8 Advance A25 X-ray diffractometer (XRD) (Bruker, Karlsruhe, Germany) was used to determine the crystal structure of CoAl-LDH@BC. Additionally, the functional groups of the CoAl-LDH@BC structure were investigated by Fourier-transform infrared spectroscopy (FTIR) (Thermo Nicolet iS5, Thermo Fisher Scientific, Waltham, MA, America), while the specific surface area and pore size distribution were characterized by a N_2_ adsorption–desorption experiment based on the ASAP2460 nitrogen adsorption–desorption isotherm analyzer apparatus (Micromeritics, Norcross, GA, America). In addition, the ESCALAB 250Xi X-ray photoelectron spectrometer (XPS) (Thermo Fisher Scientific, America) was employed to measure the valence state of CoAl-LDH@BC.

### 2.4. Experimental Method

In this work, TC with an initial concentration of 20 mg L^−1^ was selected as the target, and deionized water was employed as the experimental water throughout the research. All of the experiments were carried out in a 250 mL conical glass bottle, and the volume of the reaction solution was 100 mL. The temperature and initial pH of the reaction solution were adjusted by the water bath shaker, alongside the HCl and NaOH solutions (both 0.1 mol L^−1^). First, a TC solution (concentration of 20 mg L^−1^) was prepared in a conical bottle, followed by the addition of 30 mg and 20 mg of PMS for the oxidative degradation test. Specifically, a 0.5 mL sample was taken at certain intervals, alongside the addition of a mixture of 0.5 mL of methanol and ethanol as a quencher, and the samples were analyzed by a High-Performance Liquid Chromatograph (HPLC). Notably, the intermediate and final products of the TC degradation process were further analyzed by high-performance liquid chromatography–mass spectrometry, alongside the investigation of the effects of certain factors on the efficiency of TC degradation in the CoAl-LDH@BC/PMS reaction system, including the amount of catalyst, the amount of oxidant, the initial concentration of TC, the initial pH of solution, temperature, and common inorganic ions. In addition, free radical trapping experiments and biotoxicity tests of degraded wastewater were also carried out.

A pseudo-first-order kinetic model of Langmuir–Hinshelwood was used to analyze the degradation efficiency of TC under different conditions, in which the initial concentration of TC solution before degradation was recorded as C_0_, while the specific sampling time period was recorded as C_t_, the remaining TC content at each moment was calculated by C_t_/C_0_, K_Obs_ is the pseudo-first-order rate constant (min^−1^) and t is the reaction time (min). Based on the pseudo-first-order kinetic model of Langmuir–Hinshelwood, the pseudo-first-order kinetic constants of TC degradation efficiency of different systems were calculated by Formula (1) [25].

(1) $$\ln\left(\frac{C_{t}}{C_{0}}\right)=-K_{\mathrm{Obs}}t$$

### 2.5. Effects of TC Degraded Products on Plant Growth

Mung bean was chosen as the experimental model to determine the effects of degradation products on plant growth. Before or after the photoreaction in a Petri dish (50 mL), 40 mung bean seeds (with similar sizes) were immersed in 40 mL of deionized water, 40 mL of TC solution, and 40 mL of TC degradation product solution, respectively, followed by growing under light for 5 days at 25 °C. During the experiment, the nutrient solution was updated every day. Additionally, 40 mL of deionized water was used as a control group. After about 5 days, mung bean sprouts were harvested, the roots and stems of mung bean plants were measured.

## 3. Results

### 3.1. Characterization

**Figure 2:**
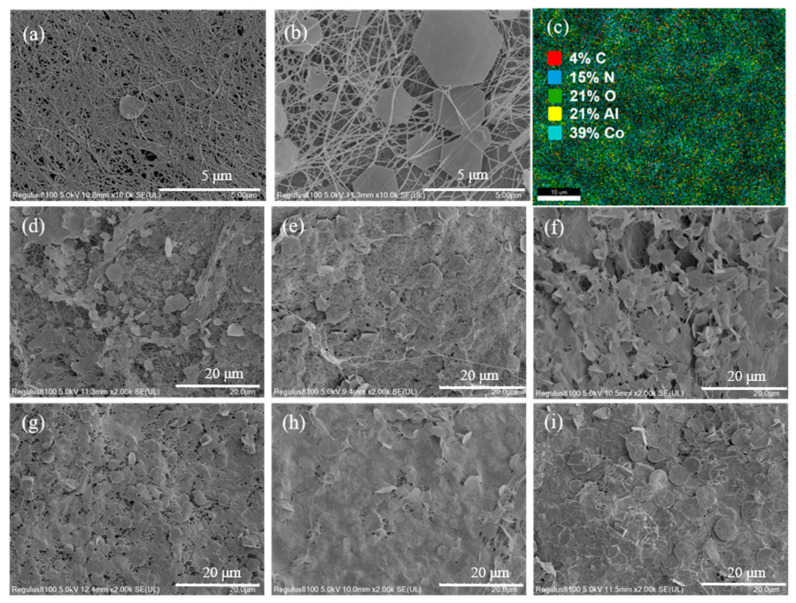
SEM images of (**a**) pure BC and CoAl-LDH@BC prepared by feeding different [Co^2+^, Al^3+^] concentrations: (**b**,**d**) 0.01-CoAl-LDH@BC, (**e**) 0.02-CoAl-LDH@BC, (**f**) 0.04-CoAl-LDH@BC, (**g**) 0.06-CoAl-LDH@BC, (**h**) 0.08-CoAl-LDH@BC, (**i**) 0.10-CoAl-LDH@BC, and (**c**) EDS mapping of 0.06-CoAl-LDH@BC.

The CoAl-LDH@BC was prepared by the in situ hydrothermal coprecipitation method with the application of BC as the template (Figure 2). Notably, BC nanofibers served as an ideal natural nanofibrous template for loading inorganic compounds or metal oxide based on their unique porous structures, substantial surface areas, and abundant surface hydroxyl groups. Hydroxyl group and ether oxygen atoms located on the BC nanofiber surfaces could adsorb Co^2+^ and Al^3+^ ions through lone pair electrons to form acid–base pairs, contributing to the formation of CoAl-LDH crystal nuclei [26]. Subsequently, the oriented growth of CoAl-LDH crystal nuclei could be promoted by the hydrothermal treatment into well-aligned lamellar sheets along the BC nanofibers. In addition, the space limitation and regulation functions of BC might partly interrupt the kinetic growth process of CoAl-LDHs, which contributed to addressing the problems of size control, uniform morphology, and ab-face stacking among CoAl-LDH sheets.

According to the SEM images, it could be found that BC exhibited a typical fibrous microstructure, and the fine fibers were coiled together in an irregular manner (Figure 2a). Meanwhile, pure BC was a translucent milky white block, and it transformed into the color of metal ions after loading hydrotalcite through the hydrothermal reaction. Additionally, CoAl-LDH@BC exhibited a three-dimensional network structure after freeze-drying, in which the three-dimensional nanostructures were formed by growing CoAl-LDH sheets on cellulose. As reported in previous studies [27,28], the LDH sheets exhibited a hexagonal structure, which was tightly attached to the cellulose by intermolecular hydrogen bonds (Figure 2b). Notably, the number of CoAl-LDH sheets in the BC structure exhibited an increasing trend (Figure 2d–i) with the increase in metal ion content in hydrotalcite solution. Most BC was relatively bare with a low concentration of hydrotalcite, and only a few were loaded with CoAl-LDH sheets (Figure 2d). Conversely, SEM images showed that a large number of CoAl-LDH sheets were stacked in the gaps of cellulose with a high concentration of hydrotalcite, and the phenomenon of fragmentation and deformation could be observed (Figure 2i). However, the overlap of excessive CoAl-LDH sheets would result in the waste of the active sites, even exhibiting negative effects on the reaction. The EDS mapping images (Figure 2c and Figure S1) displayed the uniform distribution of Co, Al, O, N, and C elements throughout the CoAl-LDH@BC. Additionally, the content of elements of Co:Al was 39:21, and it was basically consistent with the theoretical ratio (Co:Al = 2:1), which could verify the accuracy of the synthesis process.

**Figure 3:**
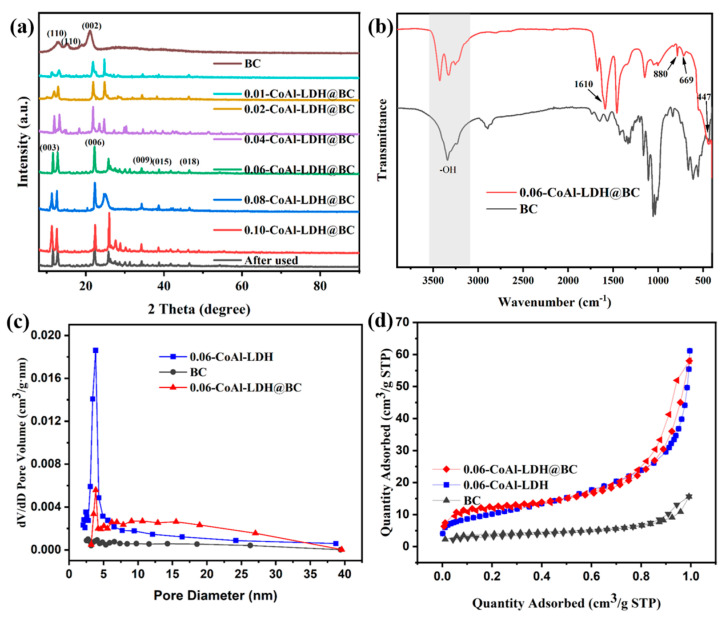
(**a**) XRD pattern, (**b**) FT-IR spectra of BC and CoAl-LDH@BC, (**c**) pore size distributions and (**d**) N_2_ adsorption–desorption isotherms.

The crystallinity and phase structure of several composites is shown in Figure 3a. According to the results, there were three characteristic peaks at 2θ = 12.9°, 15.2°, and 21.1° for Pure BC, which corresponded to the (110), (110), and (002) crystal planes of BC with a type cellulose structure [29]. For the composite CoAl-LDH@BC, compared with the standard card of CoAl-LDH hydrotalcite (PDF#00–051-0045), the diffraction peaks at 2*θ* = 11.6°, 22.2°, 34.3°, 38.7°, and 46.5° belonged to the characteristic peak of LDH, which corresponded to the (003), (006), (009), (015), and (018) crystal planes, respectively [30,31,32]. Based on the above findings, the coexistence of LDH and BC in the interior could be indicated, alongside the successful preparation of the CoAl-LDH@BC composite. In addition, the diffraction peak signal value of the composite was enhanced with the increase in LDH load, alongside the better crystallinity and a gradually weakening trend of the characteristic of cellulose. Notably, this feature was also confirmed in the SEM results. Moreover, the XRD of before and of the used 0.06-CoAl-LDH@BC composite were almost constant, revealing that the as-prepared CoAl-LDH@BC catalysts had acceptable catalytic stability.

According to the FT-IR analysis, the successful preparation of the CoAl-LDH@BC composite could be confirmed, and there were a few functional groups in this composite. In Figure 3b, there was a relatively large broad peak at 3500–3300 cm^−1^, which could be attributed to the stretching vibration of the -OH, and it mainly came from the -OH of the metal hydroxide of the main laminate and the -OH of the interlayer water molecules. In addition, the peak at 1610 cm^−1^ was caused by C=O vibrations in -COOH, while the peak at 880 cm^−1^ could be attributed to the C-H vibration [33,34]. Moreover, the peaks at 669 cm^−1^ and 447 cm^−1^ could be attributed to the vibration of Co-O and Al-O (the metal oxide in LDH), respectively. It could be found that Co^2+^ and Al^3+^ were loaded onto cellulose by binding with oxygen-containing groups on BC, which indicated the successful preparation of the CoAl-LDH@BC composite material [35].

Figure 3c,d show the N_2_ adsorption–desorption isotherm and pore size distribution for 0.06-CoAl-LDH, pure BC, and 0.06-CoAl-LDH@BC. The isotherm of 0.06-CoAl-LDH@BC showed type IV isotherm characteristics, which could indicate the existence of numerous mesoporous pores in the composite structure, and the pore size distribution mainly ranged from 2.5 nm to 15 nm. The isotherm of BC was consistent with the characteristics of a type I isotherm, and the pore size was distributed between mesoporous and microporous sizes. Table 1 displayed the specific surface area for 0.06-CoAl-LDH, pure BC, and 0.06-CoAl-LDH@BC as 36.5795, 14.6398, and 48.1334 m^2^ g^−1^, respectively. The total pore volumes of these samples were 0.0954, 0.0221, and 0.0816 cm^3^ g^−1^, respectively. The large surface area and pore volume could provide more active sites for subsequent oxidative degradation experiments.

**Figure 4:**
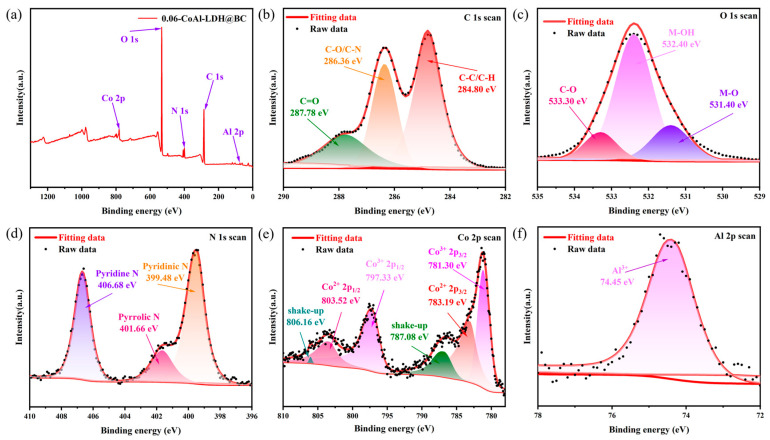
(**a**) XPS spectra of wide, (**b**) C 1s, (**c**) O 1s, (**d**) N 1s, (**e**) Co 2p, and (**f**) Al 2p of 0.06-CoAl-LDH@BC.

In order to investigate the chemical composition and valence states of the composite materials, XPS spectral analysis was performed in this study (Figure 4). According to the XPS spectrum of 0.06-CoAl-LDH@BC, there were C, O, N, Co, and Al elements in LDH@BC (Figure 4a). The C 1 s high-resolution XPS spectrum of the catalyst exhibited three peaks at 287.78 eV, 286.36 eV, and 284.80 eV, which belonged to C=O, C-O/C-N, and C-C/C-H, respectively (Figure 4b) [36,37]. The high resolution of O 1 s spectra in Figure 4c exhibits peaks at 531.4 eV and 532.4 eV which are related to lattice oxygen and hydroxide, representing the metal-O and metal-OH, respectively [38,39,40]. The high-resolution N 1 s spectrum in Figure 4d centered at 406.68, 401.66, and 399.48 eV could be assigned to the pyridine-n, pyrrolic n, and pyridinic n, respectively [15,41]. In the spectrum of Co 2p (Figure 4e), the peaks at 798.12 eV, 796.33 eV, 781.19 eV, and 785.30 eV corresponded to Co^2+^ 2p_1/2_, Co^3+^ 2p_1/2_, Co^2+^ 2p_3/2_, and Co^3+^ 2p_3/2_, respectively. Meanwhile, the peaks at 806.16 eV and 787.08 eV were shake-up peaks caused by the spin orbits of Co^2+^ and Co^3+^ [42,43,44]. These results revealed the coexistence of Co^2+^ and Co^3+^ in the catalyst, which could be transformed into each other in the REDOX reaction. In addition, only a single peak could be found in the Al 2p spectrogram (Figure 4f), which indicated that Al^3+^ was present in the LDH structure without participation in the REDOX reaction.

### 3.2. Catalytic Performance

**Figure 5:**
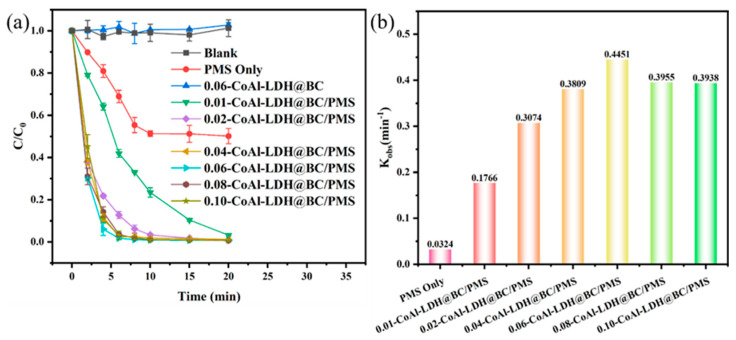
(**a**) TC removal efficiency and (**b**) Kinetic constants of TC removal in various systems (Experimental conditions: initial TC concentration of 20 mg L^−1^, PMS dosage of 20 mg, catalyst loading of 30 mg, initial pH not adjusted, room temperature 25 °C).

In order to appraise the catalytic activity of CoAl-LDH@BC composites, a degradation experiment was designed and performed in this study. As shown in Figure 5a, a single TC solution was used as a blank experiment to investigate whether TC could be self-degraded by mechanical agitation, followed by the adsorption experiment of the catalyst. It could be found that the TC content was almost unchanged during the experiment (TC content change < 5%), which indicated that TC could not degrade autonomously under natural conditions, and the adsorption amount of the catalyst itself to the target material was negligible. Only PMS and the 0.06-CoAl-LDH@BC/PMS system were added in the experiment for a control experiment, and it was found that the removal rate of TC reached 67.6% in 20 min with the addition of a single PMS. However, the removal rate of TC in the 0.06-CoAl-LDH@BC/PMS system reached 99.9% in 15 min. Although PMS was a strong oxidizing agent, no catalyst could provide an active site to produce more reactive oxygen species, which resulted in the limited removal rate of TC by a single PMS. Additionally, 0.06-CoAl-LDH@BC showed excellent catalytic performance in the experiment, alongside the great improvement in the reaction time and degradation efficiency. According to the research of the degradation efficiency of TC by a catalyst system with different metal ion contents in pre-LDH solution, the degradation rate of TC of all catalyst systems reached more than 99.7% in 20 min, and the corresponding reaction kinetic constants were shown in Figure 5b. According to the results, there were fewer LDH sheets in the catalyst with the concentration of metal ions of 0.01 M, alongside a low degradation efficiency of TC. With the continuous increase in LDH sheets until 0.06 M, the performance of the catalyst also exhibited an increasing trend. However, the degradation efficiency of TC in the 0.08-CoAl-LDH@BC/PMS system and the 0.10-CoAl-LDH@BC/PMS system was decreased. This could be attributed to the stacking of a large number of LDH sheets, which could provide more active sites. In addition, the excess free radicals would be quenched with each other [45]. In summary, based on the comprehensive consideration of the efficiency and cost of catalysts of each system, 0.06-CoAl-LDH@BC was selected for subsequent research.

**Figure 6:**
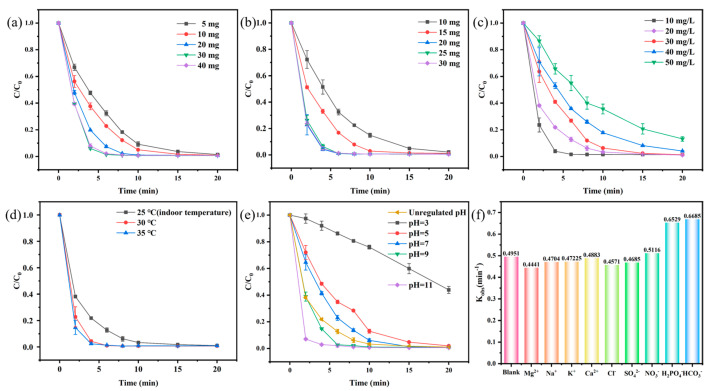
The effects of (**a**) catalyst dosage, (**b**) PMS dosage, (**c**) initial TC concentration, (**d**) reaction temperature, (**e**) initial pH, and (**f**) inorganic ion on TC degradation. (Except for the study parameters, other parameters are fixed, such as an initial TC concentration of 20 mg L^−1^, a PMS dosage of 20 mg, a catalyst loading of 30 mg, an initial pH that was not adjusted, and a room temperature of 25 °C).

As shown in Figure 6, the influence of different experimental conditions on TC degradation efficiency was explored in this study. According to the results, the degradation efficiency of TC was significantly improved with the increase in the amount of catalyst from 5 mg to 30 mg (Figure 6a), and this positive correlation could be attributed to the fact that the higher catalyst dosage could provide more active sites, thus activating the PMS and contributing to the higher TC removal efficiency. However, the degradation efficiency of TC was slightly reduced by increasing the amount of catalyst to 40 mg, which might be caused by the agglomeration caused by too much catalyst, thus reducing the number of exposed active sites [46,47]. Therefore, 30 mg of catalyst was determined as the best condition.

The effects of PMS dosage on the reaction system are displayed in Figure 6b. The degradation efficiency of TC exhibited an increasing trend with the increase in the dosage of PMS from 10 mg to 20 mg. Specifically, the addition of large amounts of PMS to the reaction solution would increase the chance of contacting the CoAl-LDH@BC active site, alongside the enhancement of the efficiency of activating PMS. However, the degradation efficiency remained unchanged when the dosage of PMS was 25 mg and 30 mg, which could result in more active sites provided by more catalysts, thus inducing the PMS to produce excess free radicals. In addition, the mutual quenching of free radicals resulted in the reduction of TC degradation efficiency [48,49], which could be attributed to the fact that a large amount of PMS competed with each other for the limited number of active sites, thus producing excessive free radicals which quench each other. Therefore, 20 mg of PMS could produce enough free radicals for the subsequent experimental condition.

When the initial concentration was high, contaminants may adhere to the surface of the catalyst, thus affecting the efficiency of the catalyst. Therefore, the effects of the CoAl-LDH@BC/PMS system on the degradation efficiency of TC with different initial concentrations was investigated (Figure 6c). Specifically, the degradation efficiency gradually decreased with the increase in the TC initial concentration from 10 mg L^−1^ to 50 mg L^−1^, which could be attributed to the limited rate of free radical production in the fixed system, and more active oxidizing substances were required with the increase in the content of the target substance. However, the degradation rates of the CoAl-LDH@BC/PMS system for a TC concentration within 10–50 mg L^−1^ could reach more than 86.8% in 20 min. Notably, at 10 mg L^−1^ TC, a degradation rate of 99.2% was achieved within 10 min.

Reaction temperature exhibits crucial functions in the TC degradation experiment, in which the room temperature was applied as a blank control. As shown in Figure 6d, each system could effectively improve the TC degradation efficiency to more than 99.9% within 15 min with the increase in reaction temperature. It could be indicated that the degradation of TC in the CoAl-LDH@BC/PMS system was an endothermic reaction, and the increase in temperature could accelerate the electron movement between the catalyst and PMS, thus improving the degradation efficiency [50,51].

The real water environment was a rather complex system, which might include a large number of organic and inorganic substances, as well as acidic and alkaline conditions. However, these characteristics would result in much trouble for the application of catalysts. Therefore, the effects of the CoAl-LDH@BC/PMS system on the degradation efficiency of TC under different initial pH values and the presence of inorganic ions in the solution were studied. As shown in Figure 6e, the degradation efficiency of the CoAl-LDH@BC/PMS system was improved under alkaline conditions. Notably, the removal rate of TC reached 97.2% within 4 min when pH = 11. This could be attributed to the fact that the high concentration of OH^−^ could promote the decomposition of PMS to produce more free radicals, thus enhancing the degradation efficiency [52]. Conversely, the removal rate of TC only reached 56.1% in 20 min when pH = 3, which could be attributed to the interference of electron transfer between the catalyst and PMS based on the hydrogen bond interaction at a high concentration of H^+^ [53]. Furthermore, acidic conditions were found to disrupt the hydroxide layer structure of hydrotalcite, leading to poor activation capability [30,54]. The electrostatic repulsion between the positively charged analytes and LDH surfaces affected the removal percentage at a pH of 3, as indicated by the pH point of zero charge (pH_pzc_ = 9.6) for LDH and the pKa values of 3.3, 7.68, and 9.68 for TC, respectively [55,56]. In general, the CoAl-LDH@BC/PMS system could effectively remove TC under the condition of pH = 5–11, which provided a wide range of choices.

Figure 6f shows the influence of several common inorganic anions (in the reaction solution) on the reaction kinetic constants of TC degradation efficiency in the CoAl-LDH@BC/PMS system. Specifically, the concentrations of Mg^2+^, Na^+^, K^+^, Ca^2+^, H_2_PO_4_^−^, SO_4_^2−^, NO_3_^−^, and HCO_3_^−^ were 117.38 mg L^−1^, 292.15 mg L^−1^, 1.45 mg L^−1^, 143.59 mg L^−1^, 460 mg L^−1^, 413.64 mg L^−1^, 117.82 mg L^−1^, and 140.58 mg L^−1^, respectively. These concentrations were consistent with the results of a water quality survey in Henan Province, China [57]. Compared with the blank experiment without the addition of inorganic ions, it could be observed from the results that all inorganic ions exhibited slight effects on the reaction system. To sum up, the CoAl-LDH@BC/PMS system exhibited a strong anti-interference ability, which could still efficiently and stably remove TC under complex environmental conditions.

**Figure 7:**
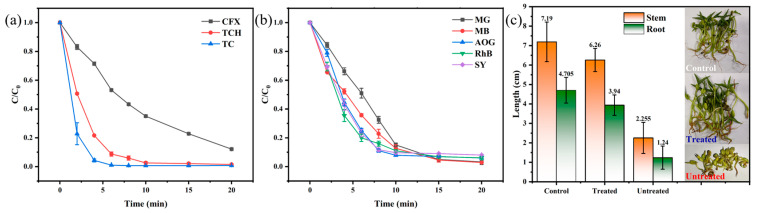
Removal efficiencies of other (**a**) antibiotics and (**b**) dyes, and (**c**) the effects of TC degraded products on plant growth (25 °C, 5 days) (Except for the study parameters, other parameters are fixed, such as initial antibiotic and dye concentrations of 20 mg L^−1^, a PMS dosage of 20 mg, a catalyst loading of 30 mg, an initial pH that was not adjusted, and a room temperature of 25 °C).

Notably, a variety of pollutants might exist in the real water environment, which was a big ordeal for the selectivity of catalyst application. Antibiotics like oxytetracycline hydrochloride (TCH) and ciprofloxacin (CFX) pose a serious threat of antibiotic resistance, while dyes represent a major industrial wastewater challenge [58,59]. Evaluating the efficiency of the prepared catalyst for TCH, CFX, and dyes allows us to demonstrate the potential real-world application of a CoAl-LDH@BC composite in treating complex wastewater streams. Therefore, the degradation efficiency of the CoAl-LDH@BC/PMS system for several antibiotics and dyes was investigated in this study. As shown in Figure 7a, the removal rates of TC and TCH in the CoAl-LDH@BC/PMS system could reach more than 99.7% and 87.9% for CFX within 20 min. The system achieved >95.7% removal for all tested dyes within 20 min, specifically 99.1% (malachite green, MG), 95.7% (methylene blue, MB), 98.4% (acid orange G, AOG), 98.7% (rhodamine B, RhB), and 95.7% (sunset yellow, SY) (Figure 7b). The CoAl-LDH@BC/PMS system exhibited high degradation efficiency for various pollutants, which could indicate that CoAl-LDH@BC was an efficient catalyst with a wide range of adaptation.

### 3.3. Toxic Test

The biotoxicity of degraded TC solution was investigated with the application of mung bean seeds as a model plant in this planting experiment (Figure 7c). Compared to the control group, the original TC solution significantly inhibited the growth of the target crop, in which the average neck length was 1.935 cm and the average root length was 1.065 cm, which could indicate the great toxicity of antibiotics. However, little difference could be seen in crop growth between the treated group (Stem = 6.775 cm, Root = 4.345 cm) and the control group (Stem = 8.025 cm, Root = 4.515 cm). In addition, the crop growth of the treatment group was even worse, indicating that the TC solution still exhibited low toxicity after the degradation of the 0.06-CoAl-LDH@BC/PMS system. This could be attributed to the fact that a small part of the intermediate products was not completely removed, and some of them remained in the solution. Based on the above findings, it could be found that the catalyst could degrade TC into small molecules with low toxicity, and there was no obvious growth effect on plants, which could confirm the good catalytic performance of the CoAl-LDH@BC composite.

### 3.4. Possible Degradation Pathway of TC

**Figure 8:**
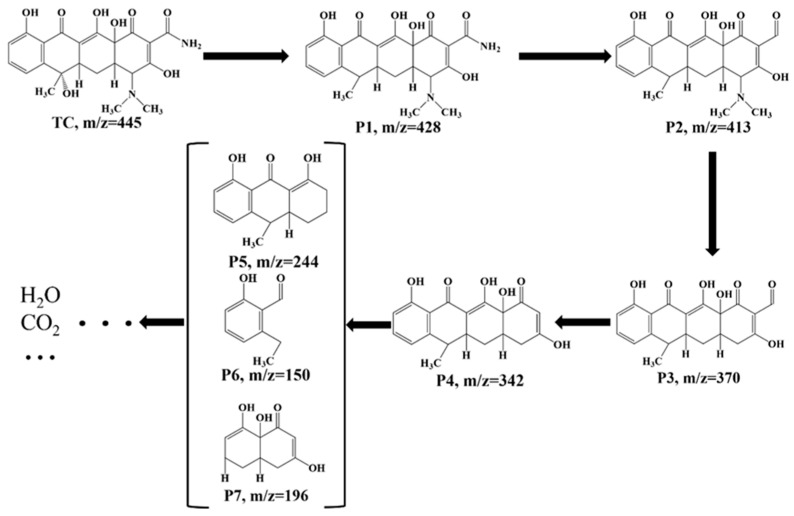
Possible degradation pathways of TC in the CoAl-LDH@BC/PMS system, and chemical structure of the intermediates. (Experimental conditions: initial TC concentration of 20 mg L^−1^, PMS dosage of 20 mg, catalyst loading of 30 mg, initial pH not adjusted, and room temperature of 25 °C).

To further elucidate the degradation pathways of TC in the CoAl-LDH@BC/PMS system, the generated intermediates were verified by a Shimadzu LC 30A HPLC coupled to a SCIEX X500R QTOF. Based on the obtained mass spectra of the intermediates (Figure S2) and previous studies [60,61], the possible TC degradation pathways are listed in Figure 8. According to the results, seven intermediate products (including *m*/*z* = 428, *m*/*z* = 413, *m*/*z* = 370, *m*/*z* = 342, *m*/*z* = 244, *m*/*z* = 196, *m*/*z* = 150) were detected, and the proposed degradation pathways were elucidated as follows. The initial degradation involved the dehydroxylation and deamination of TC molecules, yielding primary intermediates P1 and P2. Subsequently, the cleavage of the C-N bond resulted in the formation of intermediate P3, followed by the rupture of the peroxide bond, which could generate intermediate P4. Thereafter, the degradation pathway proceeded through sequential ring-opening reactions. Ultimately, the fragmented intermediates underwent complete mineralization, which converted into environmentally benign end products, including water, carbon dioxide, and other low-molecular-weight compounds.

### 3.5. Catalytic Mechanisms

**Figure 9:**
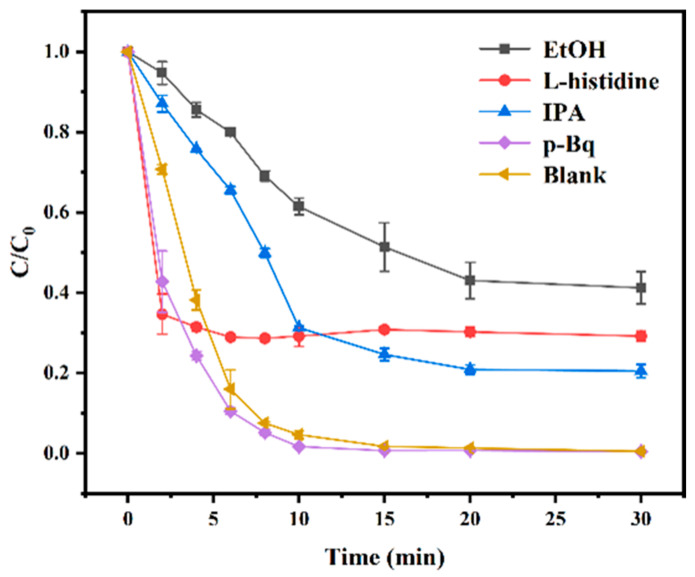
Scavenger tests with the application of ethyl alcohol (EtOH), L-histidine, propan-2-ol (IPA), and p-benzoquinone (p-Bq) as scavengers. (Except for the study parameters, other parameters are fixed: initial TC concentration of 20 mg L^−1^, PMS dosage of 20 mg, catalyst loading of 30 mg, initial pH not adjusted, and room temperature of 25 °C).

**Figure 10:**
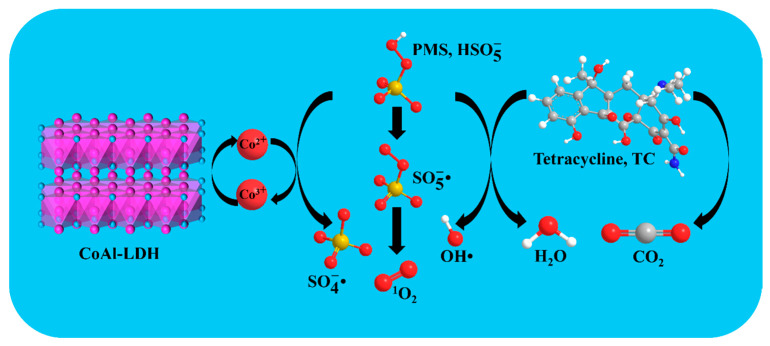
Activation mechanisms of PMS by CoAl-LDH@BC catalyst for TC degradation.

Heterogeneous catalysts could activate PMS to generate various ROS, contributing to the different mechanisms of pollutant degradation. According to some previous reports, the quenching rate constant of ethyl alcohol (EtOH) for •OH was in the range of k_•OH_ = (1.2–2.8) × 10^9^ M^−1^ s^−1^ and for${\;\mathrm{SO}}_{4}^{-}\text{•}$ was in the range of k_SO4_^−^_•_ = (1.6–7.7) × 10^7^ M^−1^ s^−1^, whereas the quenching rate constant of propan-2-ol (IPA) for •OH was in the range of k_•OH_ = (3.8–7.6) × 10^8^ M^−1^ s^−1^ [62]. Therefore, EtOH could quench both •OH and${\;\mathrm{SO}}_{4}^{-}\text{•}$ with the coexistence of the above two radicals in solution, whereas IPA could only quench •OH. In addition, L-histidine could serve as a quencher of ^1^O_2_ (k = 3.2 × 10^7^ M^−1^ s^−1^), and p-benzoquinone (p-Bq) could be applied as a quencher of ${\text{•}O}_{2}^{-}$ (k = 9.6 × 10^8^ M^−1^ s^−1^) [63,64]. As shown in Figure 9, various quenchers exhibited different effects on TC degradation in the CoAl-LDH@BC/PMS system. Compared with the blank experiment, the degradation efficiency of TC exhibited almost no difference in the presence of p-Bq, which could indicate that ${\text{•}O}_{2}^{-}$ was not produced by the active oxidizing substances in the reaction system. With the inhibition of EtOH, L-histidine, and IPA, the degradation rates of TC were 58.8%, 70.8%, and 79.5% at 30 min, respectively. This result revealed that Co^2+^ in LDH layers could activate PMS into ${\;\mathrm{SO}}_{4}^{-}\text{•}$ and •OH through a single-electron transfer process, which was the main free radical pathway for the degradation of TC by the LDH@BC/PMS system. After being reduced, Co^3+^ could generate ^1^O_2_ through a disproportionation reaction with its oxidized product ${\;\mathrm{SO}}_{5}^{-}\text{•}$. Notably, both ${\;\mathrm{SO}}_{4}^{-}\text{•}$ and •OH exhibited strong oxidizability, which could attack multiple active sites in the TC molecule (such as the benzene ring, C-N bond and methyl group), resulting in the destruction of the molecular structure of TC, alongside the final mineralization into CO_2_ and H_2_O. In addition, based on its selective oxidizing capacity, ^1^O_2_ could attack the conjugated double bond system in the TC molecule, thus enhancing the cleavage efficiency of the heterocycle and further promoting the degradation of TC. Overall, this system could realize the synergistic effects between free radicals and non-radicals, which could efficiently degrade organic pollutants, such as TC. Based on the above analysis, the possible equations in the reaction process and the schematic diagram of the catalytic mechanism were deduced as Equations (2)–(8) and Figure 10.

(2) $${\mathrm{Co}}^{2+}+{\mathrm{HSO}}_{5}^{-}\;\to {\;\mathrm{SO}}_{4}^{-}•+{\mathrm{Co}}^{3+}+{\mathrm{OH}}^{-}$$

(3) $${\mathrm{Co}}^{2+}+{\;\mathrm{HSO}}_{5}^{-}\;\to {\;\mathrm{SO}}_{4}^{2-}+{\mathrm{Co}}^{3+}+•\mathrm{OH}$$

(4) $${\mathrm{Co}}^{3+}+{\mathrm{HSO}}_{5}^{-}\;\to \;{\mathrm{SO}}_{5}^{-}•+{\mathrm{Co}}^{2+}+H^{+}$$

(5) $${\;\mathrm{SO}}_{4}^{-}•+H_{2}O\;\to {\;\mathrm{SO}}_{4}^{2-}+•\mathrm{OH}+H^{+}$$

(6) $${\;\mathrm{SO}}_{4}^{-}•+{\mathrm{OH}}^{-}\to {\;\mathrm{SO}}_{4}^{2-}+•\mathrm{OH}\;$$

(7) SO5−•+SO5−• → S2O82−+ O21

(8) SO5−•+SO5−• → 2SO4−+ O21

### 3.6. Reusability of CoAl-LDH@BC

**Figure 11:**
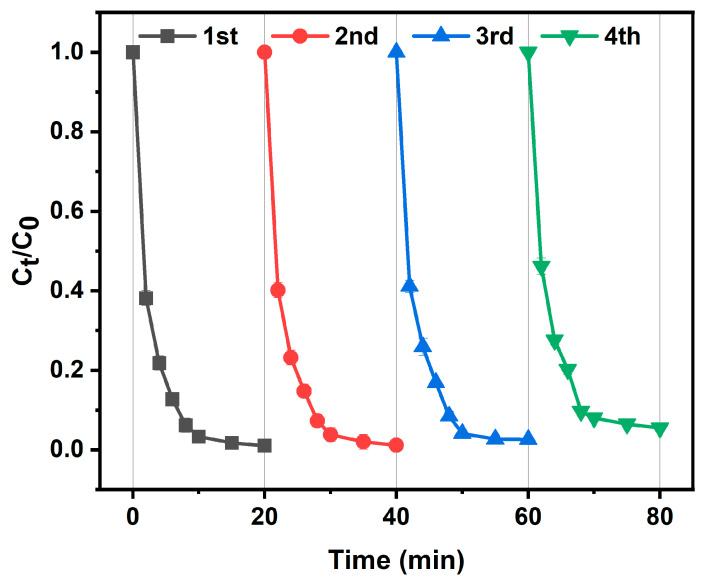
Effect of repetition times on TC degradation (Experimental conditions: initial TC concentration of 20 mg L^−1^, PMS dosage of 20 mg, catalyst loading of 30 mg, initial pH not adjusted, and room temperature of 25 °C).

To assess its practical potential, CoAl-LDH@BC’s catalyst recyclability and reusability were investigated. Figure 11 reveals a minimal reduction in degradation rate across successive cycles, yet the material’s catalytic performance stayed robust. Impressively, consistent efficiency was maintained over four consecutive uses, with 94.5% of 20 mg L^−1^ of TC degraded within 20 min. These results confirm the enduring degradation capability of CoAl-LDH@BC, underscoring its high reproducibility and reliability as a catalyst. Additionally, the post-reaction SEM images show that the catalyst’s microstructure was well-maintained, indicating remarkable structural stability (Figure S3).

Table 2 summarizes TC oxidation studies using advanced oxidation processes, along with the degradation performance of the CoAl-LDH composite for other pollutants. The CoAl-LDH@BC composite achieved remarkable catalytic efficiency (99.9% in 15 min), highlighting its practical applicability.

## 4. Conclusions

In this work, a CoAl-LDH@BC composite was successfully synthesized and employed as a PMS activator to degrade TC. Under the condition of 30 mg of catalyst dosage, 0.2 g L^−1^ of PMS concentration, and an unadjusted pH, TC (20 mg L^−1^) degradation efficiency reached 99.9% within 15 min in the CoAl-LDH@BC/PMS system. pH tests indicated that the CoAl-LDH@BC/PMS system exhibits better degradation efficiency under alkaline conditions. In addition, this system exhibited excellent performance with the coexistence of anions (Mg^2+^, Na^+^, K^+^, Ca^2+^, SO_4_^2−^, NO_3_^−^, H_2_PO_4_^−^, and HCO_3_^−^). Additionally, the toxicity of the intermediates was demonstrated to be reduced, and the degradation pathways of TC were determined based on the obtained intermediates. Quenching experiments showed that both radicals (${\mathrm{SO}}_{4}^{-}\text{•}$ and •OH) and non-radicals (^1^O_2_) were obligated in the decomposition of TC. The CoAl-LDH@BC composite exhibited excellent reusability, effectively catalyzing PMS activation for TC degradation over multiple cycles. Overall, this study introduces an environment-friendly, effective, and economical bacterial cellulose-based hydrochar catalyst, which holds great potential in the application of antibiotic removal from wastewater.

## Figures and Tables

**Table 1:** Surface area, pore size, and pore volume of various samples.

	BET Surface Area (m^2^ g^−1^)	Pore Size (nm)	V^c^ _t_ (cm^3^ g^−1^)
0.06-CoAl-LDH	36.5975	9.3998	0.0954
BC	14.6398	12.4906	0.0221
0.06-CoAl-LDH@BC	48.1334	13.2620	0.0816

**Table 2:** Comparison of the catalytic efficiency between the catalysts in this study and those reported in the literature.

Oxidizing Reagent	Pollutant	Experimental Condition	Degradation Rate (%)	Reference
N-BC@Co_3_O_4_	TC	[TC] = 10 mg L^−1^; [TC vol.] = 50 mL; catalyst = 10 mg; [PMS] = 10 mmol L^−1^; nature pH; time = 60 min	98.2	[15]
CoAl-LDH/porous g-C_3_N_4_	RhB	[RhB] = 5 mg L^−1^; [RhB vol.] = 50 mL; catalyst = 400 mg L^−1^; [H_2_O_2_] = 1.5 mL; nature pH; time = 75 min	94.5	[30]
BiOI/BiOBr	TC	[TC] = 10 mg L^−1^; [TC vol.] = 160 mL; catalyst = 0.06 g; Light= Xe lamp 300 W; pH = 8.5; time = 90 min	Completely remove	[65]
CoAl-LDH@BC	TC	[TC] = 20 mg L^−1^; [TC vol.] = 100 mL; catalyst = 30 mg; [PMS] = 20 mg; nature pH; time = 15 min	99.9	This study

## Data Availability

The original contributions presented in this study are included in the article/Supplementary Materials. Further inquiries can be directed to the corresponding author.

## Supplementary Materials

The following supporting information can be downloaded at https://www.mdpi.com/article/10.3390/biom15091283/s1: Figure S1: Images of (a) EDS analysis, and EDS mapping images of 0.06-CoAl-LDH@BC: (b) C, (c) N, (d) O, (e) Al, and (f) Co; Conditions for the LC-MS; Figure S2: Products formed by the degradation of TC by HPLC-MS; Figure S3: SEM images of 0.06-CoAl-LDH@BC after catalytic reaction.
