# Supplementary material for: In Situ Synthesis of Bacterial Cellulose-Supported CoAl-Layered Double Hydroxide as a Peroxymonosulfate Activator for Enhancing the Removal of Tetracycline
Source: Biomolecules. 2025 Sep 5;15(9):1283. doi: 10.3390/biom15091283 (PMC12467490; doi:10.3390/biom15091283)
Supplement: Supplementary file 1 [file biomolecules-15-01283-s001.zip › biomolecules-3801228-supplementary.pdf]

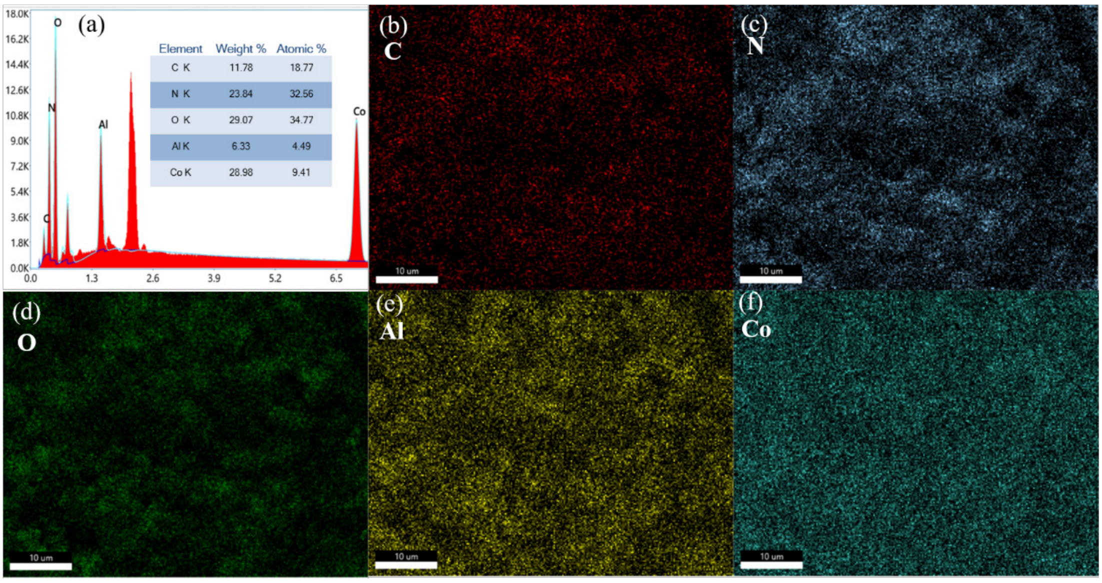

Figure S1. Images of (**a**) EDS analysis, and EDS mapping images of 0.06-CoAl-LDH@BC: (**b**) C, (**c**) N, (**d**) O, (**e**) Al, and (**f**) Co.

Conditions for the LC-MS

Analysis of TC intermediates was carried out by a Shimadzu LC 30A HPLC coupled to a SCIEX X500R QTOF packed column (C18, 150 mm× 2 mm, 3μm, Phenomenex). The LC-MS operating conditions were presented: Eluent A, formic acid solution (0.1%, V/V); Eluent B, acetonitrile (chromatographically pure); flow rate, 0.3 mL/min; column temperature, 40 °C. The gradient solvent was as followed: A=90% (0 min), 50% (2 min), 5% (6 min), 5% (8 min), 90% (10 min), 90% (12 min). Max pressure limit: 130MPa. The SCIEX X500R QTOF system with the Turbo V™ source was operated in positive electrospray ionization (ESI) mode. The TOF MS scan was conducted over a range of 100-600 m/z. Following MS parameters were selected: ion source gas one 60 psi, ion source gas1 55 psi,ion source gas 2 60 psi, curtain gas 35 psi, source temperature 550 °C, ion spray voltage 5500 V, accumulation time 0.2 s, declustering potential 70 V and collision energy 5V. An automated information dependent acquisition (IDA) approach was chosen for data collection.

Products formed by the degradation of TC by HPLC-MS were presented in Figure S2.

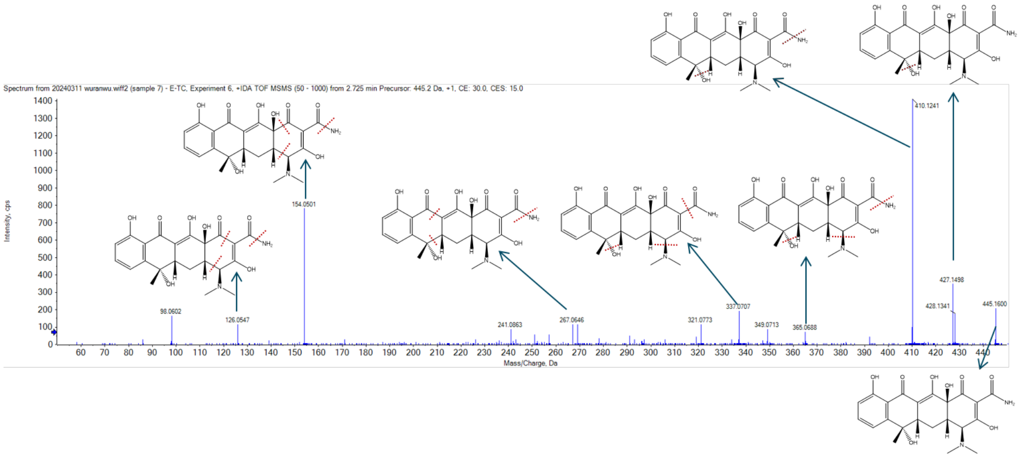

Figure S2. Products formed by the degradation of TC by HPLC-MS.

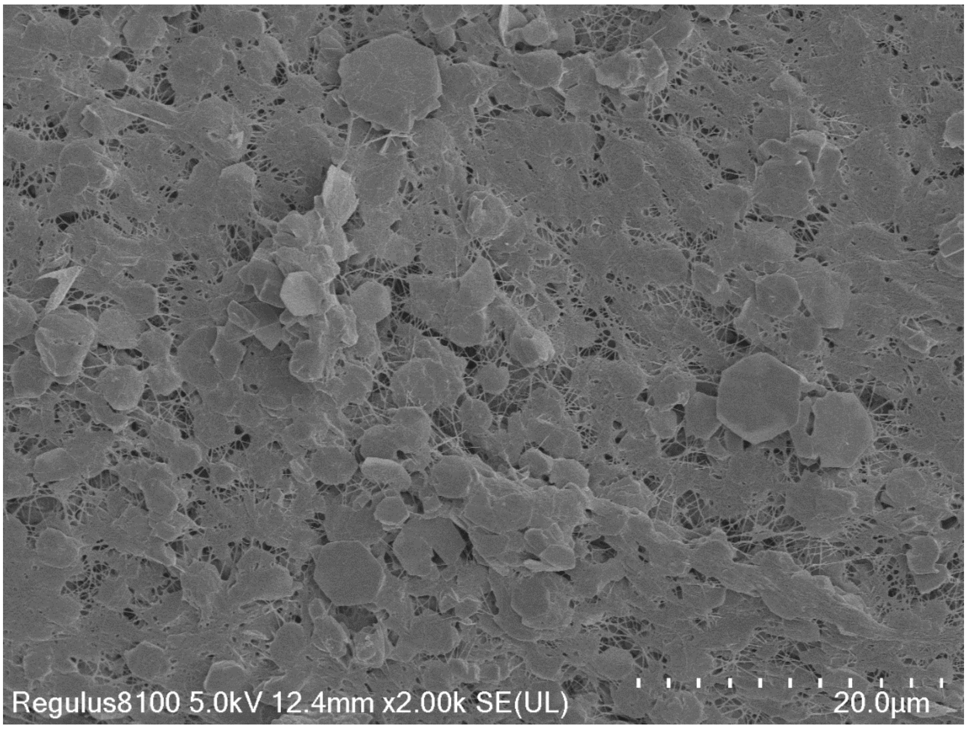

Figure S3. SEM images of 0.06-CoAl-LDH@BC after catalytic reaction.
